# Primary human mesothelial cell culture in the evaluation of the inflammatory response to different sclerosing agents used for pleurodesis

**DOI:** 10.14814/phy2.14846

**Published:** 2021-05-01

**Authors:** Michal Mierzejewski, Magdalena Paplinska‐Goryca, Piotr Korczynski, Rafal Krenke

**Affiliations:** ^1^ Department of Internal Medicine Pulmonary Diseases & Allergy Medical University of Warsaw Warsaw Poland

**Keywords:** iodopovidone, mesothelial cells, pleural effusion, pleural fluid, pleurodesis, sclerosing agents, talc

## Abstract

The mechanisms of chemical pleurodesis are still not fully explained. We aimed to evaluate the feasibility of using primary biopsy‐derived human mesothelial cells to establish an in vitro culture and to assess the response of pleural mesothelial cells to different sclerosing agents. Talc, povidone‐iodine, doxycycline, and TGF‐β were used at different doses to stimulate pleural mesothelial cells. After 6 and 24 h, mRNA expression of interleukin (IL)‐1β, IL‐6, IL‐8, TGF‐β, MCP‐1, IL‐17A, and MMP9 was measured in cultured cells, and the protein level of IL‐1β, IL‐6, and IL‐8 was measured in the culture supernatant. The most pronounced response was observed after talc exposure. It was expressed as an increase in IL‐1β concentration in culture supernatant after 24 h of higher talc dose stimulation compared to 6 h of stimulation (17.14 pg/ml [11.96–33.32 pg/ml] vs. 1.84 pg/ml [1.81–1.90 pg/ml], *p* = 0.02). We showed that culture pleural mesothelial cells isolated from pleura biopsy specimens is feasible. Inflammatory responses of mesothelial cells to different sclerosants were highly variable with no consistent pattern of mesothelium reaction neither in terms of different sclerosing agents nor in the time of the most significant reaction. We demonstrated that pro‐inflammatory mesothelial response includes an increase in IL‐1β mRNA expression and protein production. This may suggest the role of IL‐1β in the formation and maintenance of the inflammatory response during pleurodesis.

## Graphical abstract

We performed the prospective study using primary, biopsy‐derived human mesothelial cells to establish an in vitro culture and to assess the response of pleural mesothelial cells to four different sclerosing agents (talc, povidone‐iodine, doxycycline, and transforming growth factor β) in different doses. Our study shows, that it is feasible to culture pleural mesothelial cells isolated from pleura biopsy specimens, that can be used for research on pleurodesis mechanisms. We found that pro‐inflammatory mesothelial response includes an increase in interleukin (IL)‐1β mRNA expression and protein production, which suggests the role of IL‐1β in the formation and maintenance of the inflammatory response during pleurodesis.

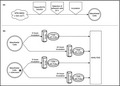

## 1 INTRODUCTION

Pleurodesis is a well‐established definitive treatment option for patients with recurrent malignant pleural effusion (MPE) (Bibby et al., 2018). Its essence is stimulation of adhesion formation between the visceral and parietal pleura in order to obliterate the pleural cavity and to prevent fluid reaccumulation. Intrapleural instillation of chemicals (chemical pleurodesis) has long been the major intervention to create parietal‐visceral symphysis. In recent years, a second definitive therapeutic option for MPE has been successfully developed. This pertains to the installation of the indwelling pleural catheter (IPC). Interestingly, it has been demonstrated that long‐standing pleural drainage is associated with autopleurodesis which enables IPC removal with no pleural fluid recurrence (Davies et al., 2012; Muruganandan et al., 2018; Wahidi et al., 2017). Thus, a new idea has emerged to apply IPC together with sclerosants or sclerosant‐coated IPC (Bhatnagar et al., 2018). Nonetheless, chemical pleurodesis remains one of the main therapeutic methods for patients with symptomatic and recurrent MPE. Albeit the concept of chemical pleurodesis is convincing and the history of its use is relatively long (since 1901; Spengler, 1906), the knowledge about the mechanisms of pleurodesis is highly incomplete. Over several decades, various sclerosants have been used, including talc (Bethune, 1935), antibiotics (doxycycline; Salomaa et al., 1995), antiseptics (silver nitrate; Bucknor et al., 2015, iodopovidone; Caglayan et al., 2008), cytostatic agents (bleomycin; Lynch et al., 1996), radioactive colloidal gold (Botsford, 1964) transforming growth factor β (TGF‐β; Light et al., 2000) autologic blood (Keeratichananont et al., 2015) or even bacteria (*Streptococcus* *pyogenes* A3 [OK‐432]; Luh et al., 1992) With the exception of TGF‐β, most of the above sclerosants act as local irritants and pro‐inflammatory stimuli (Light et al., 2000).

Different cells and biochemical pathways are involved in the formation of pleural adhesions during pleurodesis. Production and release of cytokines, as well as adhesion molecules leading to activation of the coagulation cascade and an imbalance between fibrinolysis and fibrinogenesis (in favor of the latter), are construed as the most important pathways leading to pleurodesis.

Mesothelial cells are believed to be the main structural axis of the process. These cells have been shown to secrete a variety of mediators in response to sclerosing agents (Rodriguez‐Panadero & Montes‐Worboys, 2012). Subsequently, other cell populations including neutrophils, fibroblasts, and macrophages are involved. IL‐8 and neutrophils seem to be critically important in the initiation and maintenance of pleural inflammation (Psathakis et al., 2006) Numerous other mediators have also been shown to play a role in this process, including TGF‐β, basic fibroblast growth factor (bFGF), and pro‐inflammatory cytokines IL‐6, IL‐1 (Mierzejewski et al., 2019).

The aim of the study was to evaluate the inflammatory response of primary mesothelial cells to different sclerosing agents (talc, povidone‐iodine, doxycycline, and TGF‐β) and to assess the relationship between sclerosant concentration as well as duration of stimulation and the level of pro‐inflammatory cytokines expression.

## 2 MATERIAL AND METHODS

### 2.1 General study design

**FIGURE 1:**
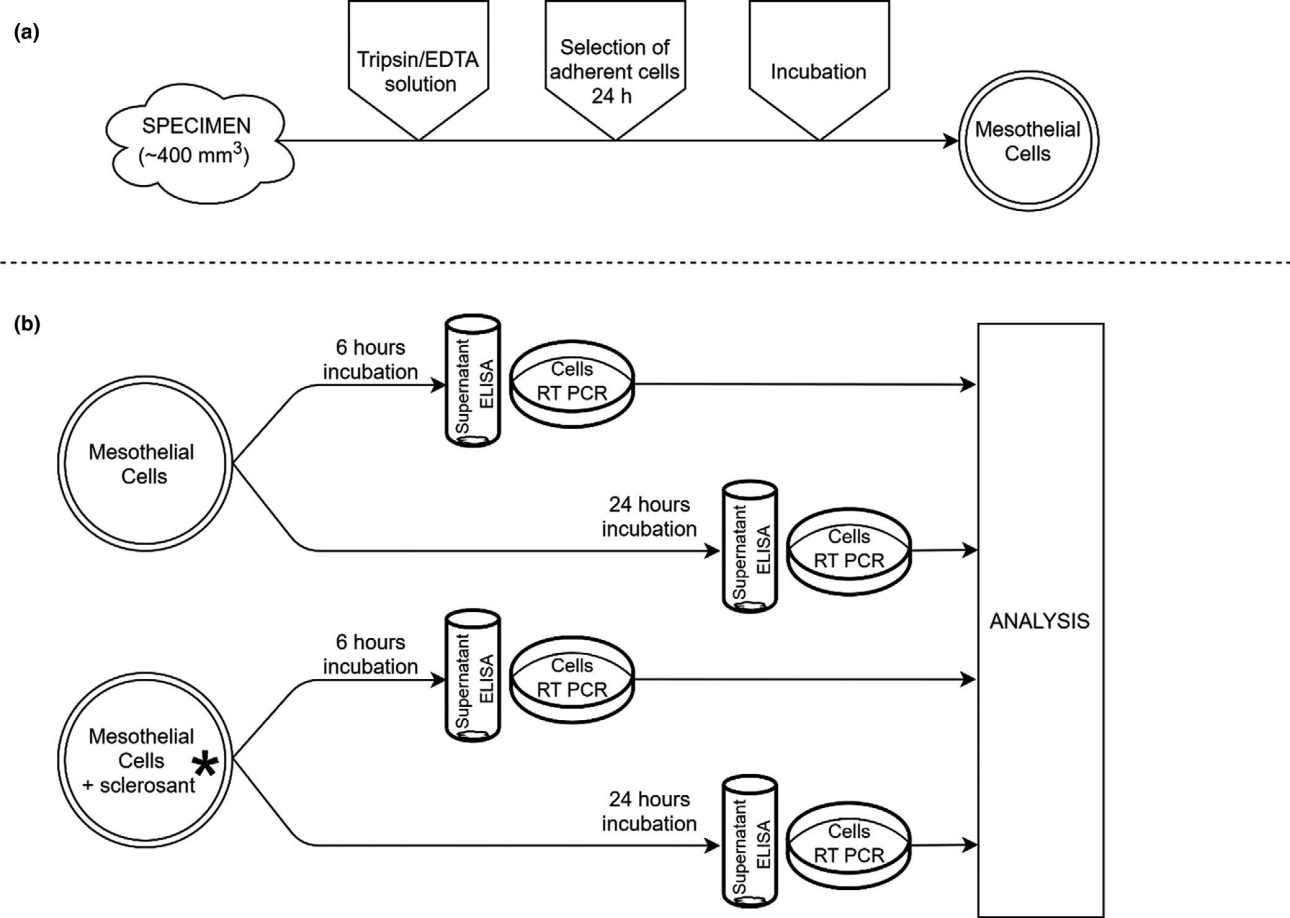
Simplified design of the study with two major steps: (a)—isolation and culture of pleural mesothelial cells, and (b)—stimulation of pleural mesothelial cells with four sclerosants in two different doses and analysis of the results.*Experiments were repeated for each of the following: Talc (2 and 20 μg/ml), povidone‐iodine (0.01% and 0.001%), doxycycline (0.2 and 2 μg/ml), and TGF‐β (0.2 and 2 μg/ml)

This was an experimental study based on mesothelial cell culture. Normal human pleural mesothelial cells were isolated from pleura specimens obtained from patients with no history of pleural diseases undergoing scheduled coronary artery bypass surgery. All patients signed an informed consent for pleural sampling. Small pleural biopsies (~400 mm^3^) were taken by a skilled cardiac surgeon and the procedure did not impact the time or the extent of the planned surgery. The protocol of the project was approved by the institutional Bioethics Committee (KB/214/2017). The major steps of the study are depicted in Figure 1.

### 2.2 Mesothelial cell isolation and culture

Pleural samples from four patients were used to isolate pleural mesothelial cells. The pleural biopsies were cut into smaller pieces, washed with PBS, and cell isolation was performed with 0.125% trypsin and 0.01% tethylenediaminetetraacetic acid (EDTA) treatment for 30 min at 37°C in 5% CO_2_ as previously described (Stylianou et al., 1990; Yung et al., 2006) Cell suspensions were centrifuged (300 *g*, 10 min, room temperature). The cell pellets were resuspended in a final total volume of 5 ml of mesothelial growth M199 medium (Sigma‐Aldrich, St. Louis, MO, USA) supplemented with 3.3 nM mouse epidermal growth factor (Corning, Corning, NY, USA), 400 nM hydrocortisone (Sigma‐Aldrich), 870 nM zinc‐free bovine insulin (Sigma‐Aldrich), 20 mM 4‐(2‐hydroxyethyl)‐1‐piperazineethanesulfonic acid (HEPES;​ Gibco, Thermo Fisher,Waltham, MA, USA), 0.001% Trace elements (Corning) containing 1% antibiotic cocktail (Gibco, Thermo Fisher) and seeded into sterile plastic T25 bottle. Cells were incubated in a plastic dish for 24 h at 37°C in 5% CO_2_. The undetached cells were removed and the cell medium was changed. The adhered cells were labeled as first passage. At 80% of confluency, cells were detached with Trypsin‐EDTA solution and cultured in 24‐well plates for the experimental phase. One cell line from one subject with the best viability was selected for further experiments. The further steps of the study were performed on two to three passages.

### 2.3 Human lung fibroblast culture

Human lung fibroblasts (CC‐2512; Lonza) were cultured in minimum essential medium (MEM) (Gibco, Thermo Fisher) supplemented with 1% MEM non‐essential amino acids (Gibco, Thermo Fisher).

### 2.4 Flow cytometry analysis for cell phenotyping

Cells were stained with antibodies against the following surface‐binding molecules: CD45 PE (clone 2D1; eBioscence Thermo Fisher Scientific), CD14 Alexa Fluor 488 (clone M5E2; BD Biosciences), CD71 Alexa Fluor 700 (clone M‐A712; BD Biosciences) CD90 PE‐CF‐594 (clone 5E10; BD Biosciences) and incubated for 20 min in the dark at room temperature. Mesothelial cells were identified using CD71 expression as an identity marker based on previous research by Kienzle et al. (2018). Fibroblasts were identified using CD 90 expression as an identity marker based on previous research by Kisselbach et al. (2009).

Cells were analyzed by flow cytometry using a FACSCelesta instrument (BD Biosciences) equipped with blue (488 nm), violet (405 nm), and red (640 nm) lasers. Unstained cells and compensation beads (BD Biosciences) were used to set voltages and create single‐stain‐negative and ‐positive controls. Compensation was set to account for spectral overlap between the seven fluorescent channels used in the study. Samples were examined by side scatter area versus forward scatter area (FSC‐A), then using forward scatter height (FSC‐H) versus FSC‐A to select single cells, eliminating debris, and clumped cells from the analysis.

### 2.5 Interventions and measurements in the mesothelial cell line

Talc (2 and 20 μg/ml; Novatech), povidone‐iodine (PVP‐I; 0.01% and 0.001%; ChemCruz), doxycycline (0.2 and 2 μg/ml; Sigma‐Aldrich), and TGF‐β (0.2 and 2 μg/ml; R&D Systems) were used as sclerosing substances. The cells treated with sclerosants were cultured for 6 and 24 h. At these two specific time points, mRNA expression as well as the protein levels of selected cytokines and growth factors were measured in mesothelial cells and culture supernatants, respectively (Figure 1). The study included the measurements of mRNA for the following mediators: IL‐1β, IL‐6, IL‐8, TGF‐β, MCP‐1, IL‐17A, and MMP9. This was supplemented by the assessment of IL‐1β, IL‐6, and IL‐8 protein concentrations. Four independent experiments were performed in triplicates (*n* = 4).

#### 2.5.1 Protein concentration measurement

The levels of IL‐1β, IL‐6, and IL‐8 in cell culture supernatants were measured using enzyme‐linked immunosorbent assays (ELISA kits; Thermo Fisher) according to the manufacturer's instructions. The sensitivity of the applied kits was 2 pg/ml.

#### 2.5.2 RNA isolation and reverse transcription

Total RNA was isolated from the cells using Trizol (Sigma‐Aldrich). The concentration and purity of isolated RNA were measured on a DU650 spectrophotometer (Beckman Coulter). One microgram of total RNA was used for reverse transcription (Thermo Fisher).

#### 2.5.3 Real‐time PCR

Real‐time polimerase chain reaction (PCR) was performed with an ABI‐Prism 7500 Sequence Detector System (Applied Biosystems). For PCR reaction 0.8 µl of cDNA was amplified in 16 µl PCR volume, containing a Power SYBR Green PCR mastermix (Thermo Fisher) with 150 nM of specific primers (Thermo Fisher, BioRad). The sequence of the primers is shown in Table 1. 18s rRNA was applied for each sample as an endogenous gene in order to normalize gene expression levels. Unstimulated cells from each experiment were used as a calibrator. Each sample was measured in duplicate. Relative quantification values were calculated by the 2^−∆∆CT^ method. The results were expressed as relative quantification units (fold change).

**TABLE 1:** Sequence of primers used in PCR

	Forward primer	Reverse primer	Product size
18s rRNA	GGATGAGGTGGAACGTGTGAT	AGGTCTTCACGGAGCTTGTTG	148
IL‐6	CCGGGAACGAAAGAGAAGCT	GCGCTTGTGGAGAAGGAGTT	67
IL‐8	GAGCACTCCATAAGGCACAAACT	ATCAGGAAGGCTGCCAAGAG	149
MMP9	GCTCACCTTCACTCGCGTG	CGCGACACCAAACTGGATG	61
MCP‐1	TCTGCCCGCTTTCAATAAGAG	GTGCGAGCTTCAGTTTGAGAATT	97
TGF‐β	CAGCAACAATTCCTGGCGATA	AAGGCGAAAGCCCTCAATTT	136
IL‐17A	AGGAATCACAATCCCACGAAAT	GGTGAGGTGGATCGGTTGTAGT	149
IL‐1β	qHsaCIP0033362		119

### 2.6 Statistical analysis

Statistical analyses were performed using Statistica 12.0 software (StatSoft Inc.). The technical replicates were averaged for four independent experiments on pleural cells isolated from one subject. The normality of data distribution was tested using Shapiro–Wilk test. Differences between continuous variables were tested using nonparametric Mann–Whitney *U* test. Data are presented as median and interquartile range. Differences were considered statistically significant at *p* < 0.05.

## 3 RESULTS

### 3.1 Cell phenotyping

**FIGURE 2:**
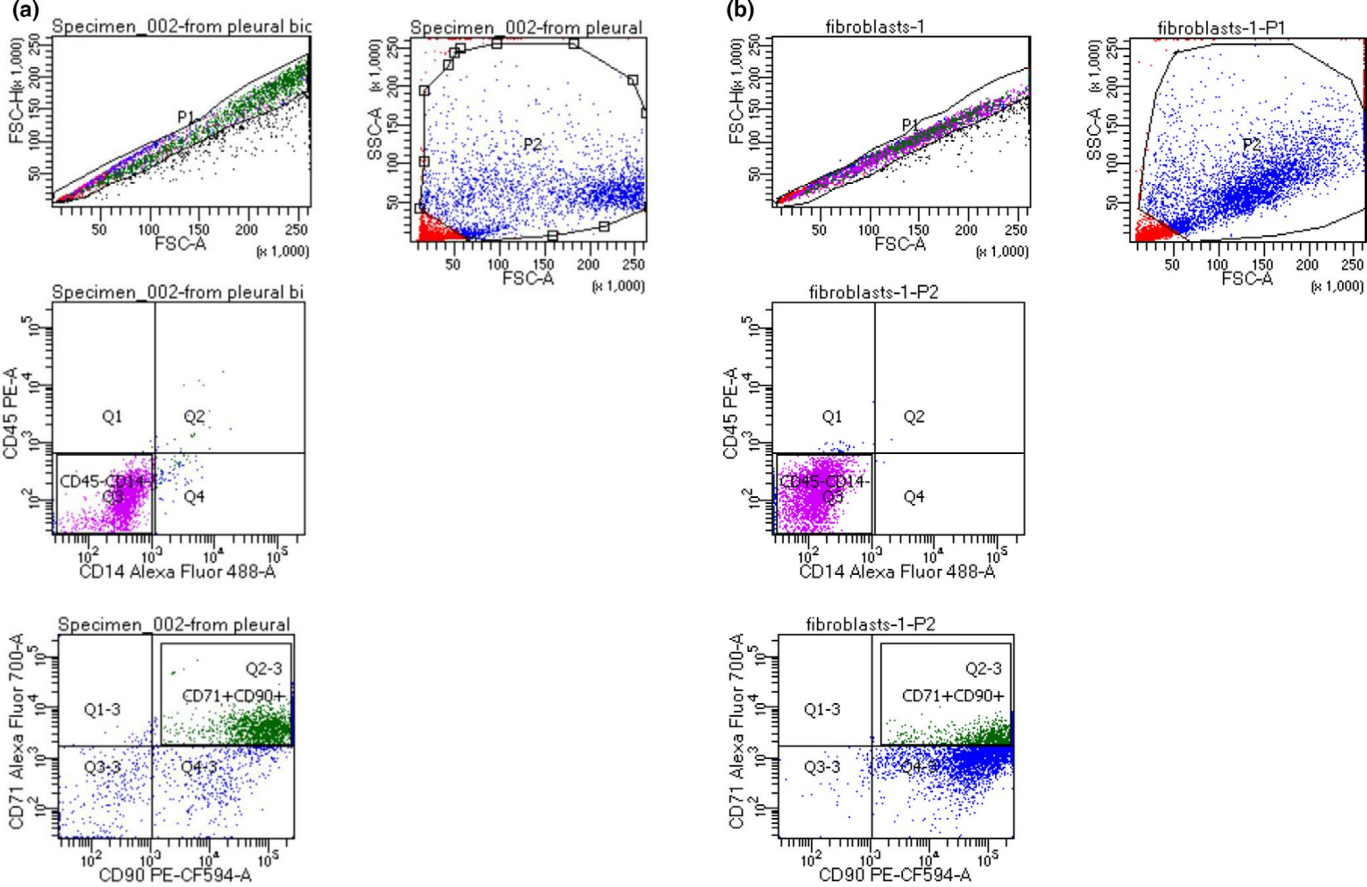
Gating strategy for (a)—identification of cells isolated from pleural biopsies, (b)—comparison with lung fibroblasts (Lonza)

The cells isolated from pleura were identified as CD45−CD14− cells. This result implied no leukocyte/macrophage contamination. The majority of the cells isolated from pleural biopsies were identified as CD45−CD14−CD90+CD71+ (mesothelial cell phenotype, 67.5%) in contrast to lung fibroblasts (cell line CC‐2512; Lonza) where the contribution of CD45−CD14−CD90+CD71+ cells was 17.5% (Figure 2). Hence we were able to demonstrate that the majority of cells in the examined cell cultures had features of the mesothelial phenotype.

### 3.2 mRNA expression of mediators in mesothelial cells

There were variable responses of mesothelial cells to stimulation with different sclerosing agents. Six hours after sclerosant application only MMP‐9 mRNA expression changed significantly in cultures treated with the higher PVP‐I doses (0.01%); (0.036 fold change [0.017–0.062 fold change]) compared to controls (1.15 fold change [1.14–5.31 fold change]), *p* = 0.02. Regardless of the sclerosing agent and its dose, no significant increase in mRNA expression for the remaining cytokines was demonstrated after 6 h of exposure.

**FIGURE 3:**
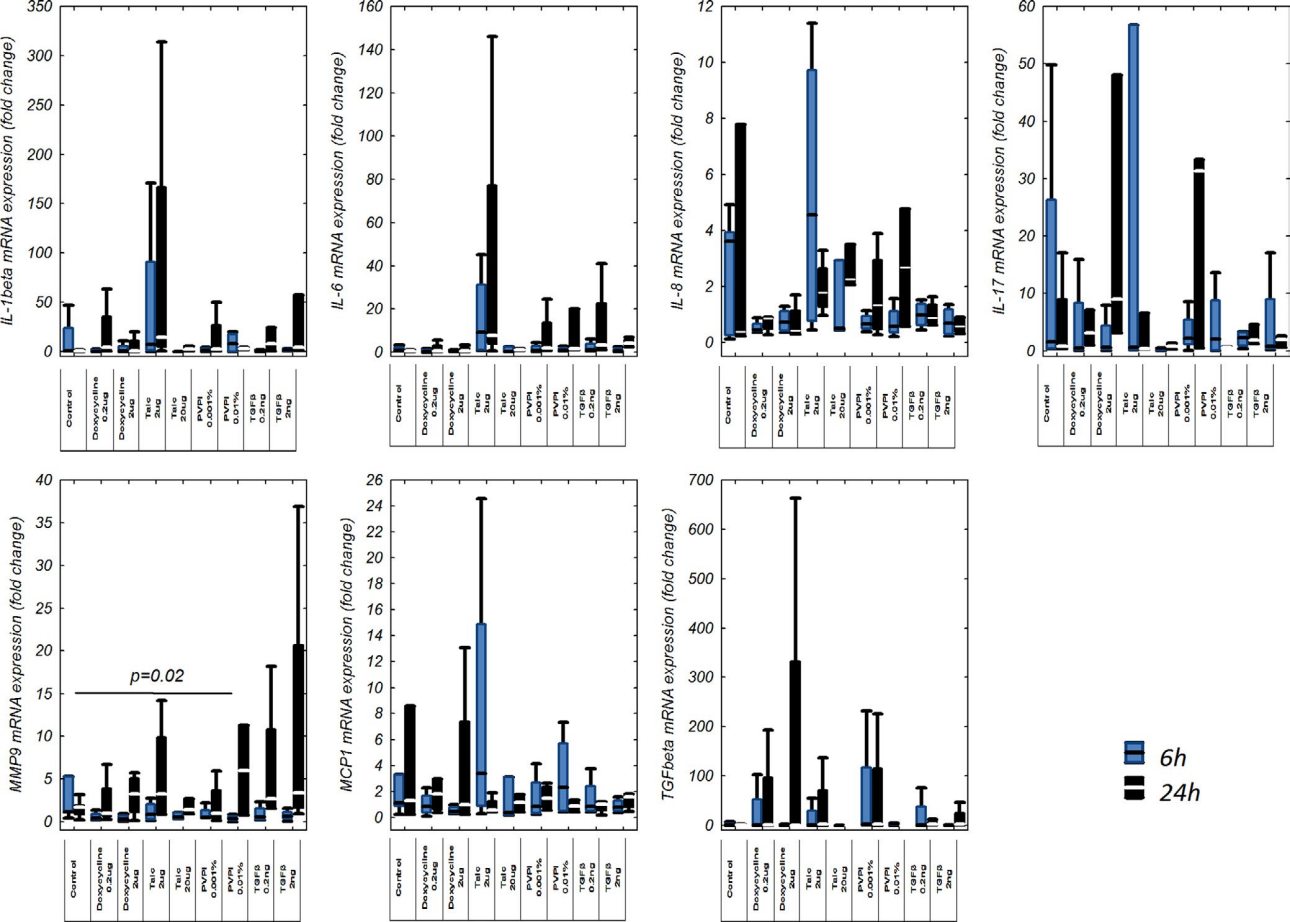
IL‐1β, TGF‐β, IL‐6, MMP9, IL‐8, MCP‐1, and IL‐17 mRNA expression in cultured mesothelial cells stimulated with different sclerosants (doxycycline, PVP‐I, talc, and TGF‐β) for 6 or 24 h. Four independent experiments were performed in averaged triplicates (*n* = 4) on second or third passage of cells from one subject propagated and divided into further cultures from passage 1. IL, interleukin; MMP9, matrix metalloproteinase 9; MCP‐1, monocyte chemoattractant protein; PVP‐I, iodopovidone; TGF‐β, transforming growth factor β

The changes found 24 h after sclerosant application were highly heterogeneous with an increase in mRNA expression for various mediators after stimulation with different sclerosants, however, none of them reached statistical significance (Figure 3).

Generally, mRNA expressions of evaluated mediators tended to be higher after 24 h of stimulation and with higher doses of sclerosants. This tendency can be most clearly seen in the IL‐8 and MMP‐9 expressions, however, they were not statistically significant (Figure 3).

### 3.3 IL‐1β, IL‐6, and IL‐8 protein level

Application of talc was associated with the most significant changes in cytokine concentrations.

Treatment with the lower talc dose (2 μg/ml) resulted in an insignificant increase in IL‐1β protein after 6 and 24 h versus their controls (4.02 pg/ml [4.05–11.28 pg/ml] vs. 4.36 pg/ml [2.65–5.78 pg/ml] and 19.53 pg/ml [13.13–29.90 pg/ml]) vs. 4.31 pg/ml [2.91–8.21 pg/ml]).

Exposure of mesothelial cells to the higher talc dose (20 μg/ml) caused a significant decrease in IL‐1β production after 6 h (1.85 pg/ml [1.81–1.90 pg/ml], *p* = 0.02) and insignificant increase as after 24 h (17.14 pg/ml [11.9–33.32 pg/ml]) versus controls. An IL‐1β concentration in culture supernatant after 24 h of higher talc dose stimulation was significantly higher compared to 6‐h stimulation (*p* = 0.02).

Mesothelial cells produced a significantly lower level of IL‐1β after 6 h of treatment with 20 μg/ml when compared to 2 μg/ml dose of talc after 6 h of exposure (*p* = 0.008).

The concentrations of IL‐6 after 6 h of stimulation with different sclerosants were similar to those found in the control group. There was a trend to increase IL‐6 levels in cultures stimulated with talc, doxycycline, PVP‐I, and TGF‐β (in both doses) after 24 h of exposure compared to cell cultures without stimulation and the cultures stimulated for 6 h, but the differences were not statistically significant.

**FIGURE 4:**
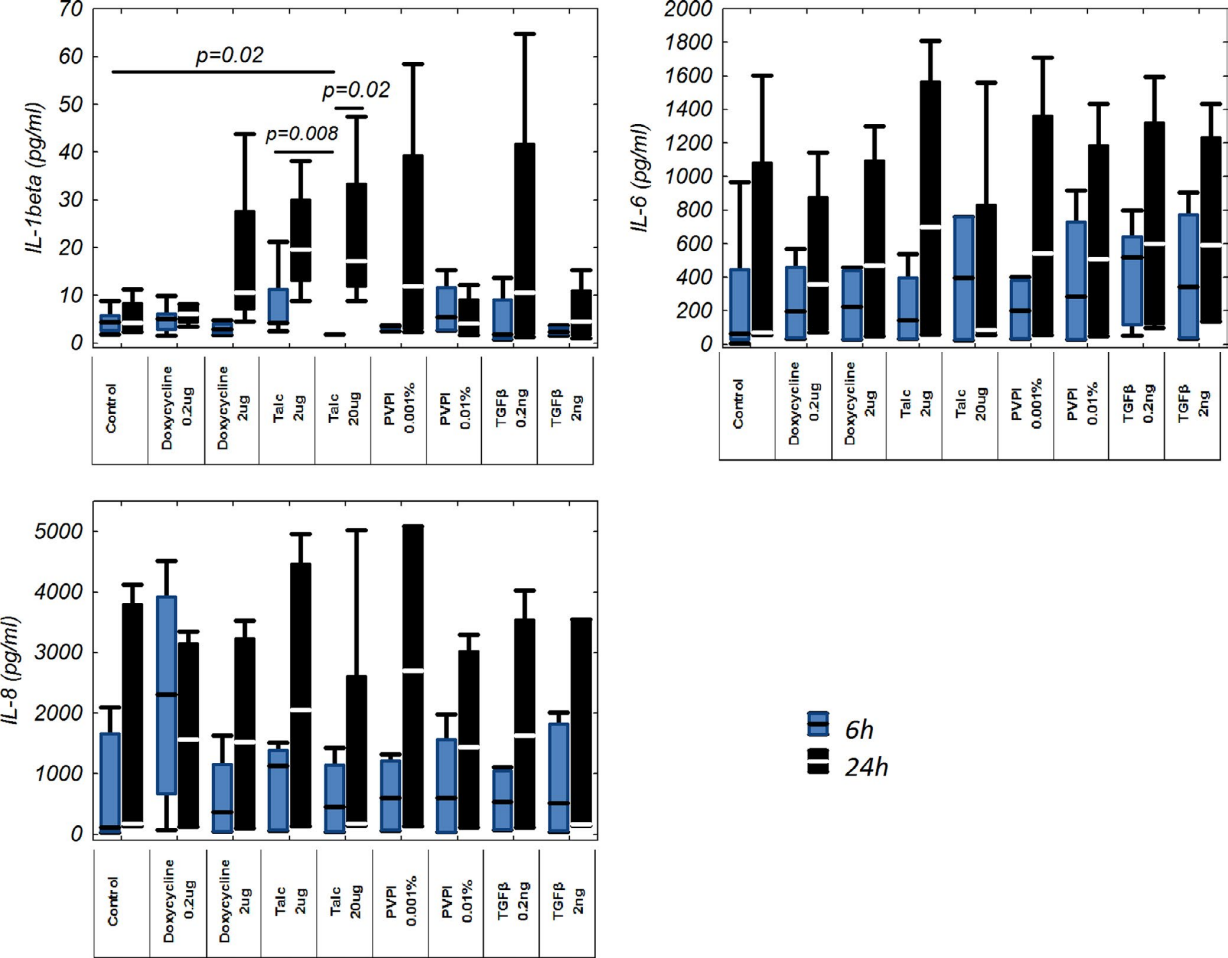
IL‐1β, IL‐6, and IL‐8 protein levels in mesothelial cell culture supernatants stimulated with different sclerosants (doxycycline, PVP ‐ I, talc, and TGF‐β) for 6 or 24 hours. Four independent experiments were performed in averaged triplicates (n=4) on 2nd or 3rd passage of cells from one subject propagated and divided into further cultures from passage 1. IL‐1, IL‐6, IL‐8, interleukin 1, 6 and 8; PVP‐I, iodopovidone; TGF‐β, transforming growth factor β

The pattern of changes in IL‐8 concentration was similar to that found for IL‐6. The concentration of IL‐8 after 6 h of stimulation did not significantly change in any sclerosant group. Higher levels of IL‐8 were found in all cultures 24 h after the exposure (except the culture with the lower dose of doxycycline) compared to unstimulated cells and IL‐8 levels measured 6 h after the exposure. These differences, however, did not statistical significance (Figure 4).

## 4 DISCUSSION

Our study showed that normal, primary mesothelial cells isolated from patients undergoing cardiac surgery can be successfully used to evaluate the effect of different stimuli. We found a diversity of effects produced by various sclerosing agents applied to mesothelial cell cultures. The most pronounced effect of talc was demonstrated for IL‐1β. This refers to an increased IL‐1β level in mesothelial cell culture supernatant. Overall, a 24‐h exposure of mesothelial cells to different sclerosing agents resulted in a more conspicuous increase in inflammatory or profibrogenic mRNA and protein concentration than a 6‐h exposure. However, there were some exceptions, including IL‐8, MCP‐1, and IL‐17A (mRNA) concentration after the exposure to the lower talc dose, as well as MCP1 mRNA after a higher dose of PVP‐I exposure. Hence, it must be admitted that the changes in mRNA expression and cytokine concentration resulting from mesothelial cell exposure to different doses of various sclerosants were highly heterogeneous.

We believe, our preliminary study provides a solid base for further evaluation of isolated mesothelial cell response to various sclerosing agents. Although the active role of pleural mesothelial cells in pleurodesis has been earlier postulated, data supporting this view mainly come from animal models and in vitro studies on commercially available immortalized human pleural mesothelial cells. In this context, it should be underlined that detailed biochemical in vitro responses of human primary pleural mesothelial cells were insufficiently evaluated. In fact, this is one of the few basic studies that used in vitro patient mesothelial cell cultures. Furthermore, the data on the precise mechanisms of pleurodesis using other agents than talc are very scarce. To our knowledge, there were no previous studies to compare the inflammatory response of mesothelial cells to various different sclerosants and their different doses. Thus, this is the first study which showed a different potency of pro‐inflammatory response of mesothelial cells to different doses of doxycycline, iodopovidone, talc and TGF‐β after 6 or 24 h, measured as IL‐1β, TGF‐β, IL‐6, MMP9, IL‐8, MCP‐1, and IL‐17 mRNA expressions and IL‐1β, IL‐6, and IL‐8 protein levels.

The highest mesothelial response in our study was associated with talc exposure and was expressed as an elevation of IL‐1β concentration in culture supernatant. Since previous studies reported neutrophils, monocytes (Rodriguez‐Panadero & Montes‐Worboys, 2012) and macrophages (Akexandrakis et al., 2000) as the main sources of IL‐1β in MPE (also after pleurodesis), an increased level of IL‐1β in the monoculture of mesothelial cells was a rather unexpected finding. Interestingly, although the role of IL‐1β in pleural inflammation, for example, in pleural empyema (Silva‐Mejías et al., 1995) and in pleurisy model that used carbon nanotubes (Murphy et al., 2012) has been extensively evaluated, its secretion by pleural mesothelial cells in the context of pleurodesis has not been studied so far. Our study demonstrated that mesothelial cells may be a possible additional source of IL‐1β. Albeit mesothelial cells constitute only a small percentage of pleural fluid cells (Noppen, 2004), they form a continuous internal layer of both visceral and parietal pleura. Thus, the role of these cells as the source of pleural IL‐1β cannot be underestimated. The results of our study suggest a possible utility of IL‐1β as a potent marker of pro‐inflammatory and perhaps profibrogenic activity of mesothelial cells.

Interleukin‐1β is known to release plasminogen activator inhibitor 1 (PAI‐1) and TGF‐β from pleural mesothelial cells. As PAI constrains urokinase and tissue plasminogen activator (tPA) activity (which both convert plasminogen into plasmin), its elevated level results in reduced intrapleural fibrinolysis. An enhanced organization of fibrin (the final product of the coagulation cascade) leads to the formation of delicate bonds between the visceral and parietal pleura. This phenomenon was demonstrated by Tucker et al. (2014) in an animal model. Meanwhile, the secretion of TGF‐β and other growth factors (e.g., PDGF, bFGF) by IL‐1β‐stimulated pleural mesothelial cells leads to the recruitment and proliferation of fibroblasts, triggering the fibrosis pathway (Schwarz & Star, 2012). In contrast, it has been shown that IL‐1β concentration in pleural fluid correlated negatively with pleural fluid tPA levels (Cl et al., 2005). That may suggest that IL‐1β is an important player in inflammation, fibrosis, and coagulation pathways activated in the process of pleurodesis (Mierzejewski et al., 2019). Pro‐inflammatory mesothelial response manifested by tendency in increasing IL‐1β protein production found in our study seems to be in line with the above considerations and suggests the role of IL‐1β in the formation and maintenance of the inflammatory response in the course of pleurodesis.

We did not observe a significant increase in IL‐6 and IL‐8 protein production by mesothelial cells treated with different sclerosants. This seems somewhat surprising, as these cytokines are construed as classical inflammatory mediators. Under normal conditions, IL‐8 is produced by mesothelial cells and its production significantly increases in response to inflammatory stimuli (Light & Gary Lee, 2003). IL‐8 induces neutrophil influx to the pleural cavity (Psathakis et al., 2006), and it can be blocked by anti‐IL‐8 antibodies (Broaddus et al., 1992). Neutrophils migrating to the pleura produce and release a variety of other cytokines responsible for maintaining an already activated inflammatory pathway (Rodriguez‐Panadero & Montes‐Worboys, 2012). The role of IL‐8 in pleurodesis as evaluated in an animal study by Marchi et al. (2006) who injected talc to rabbit pleura and collected pleural fluid after 6, 24, or 48 h. In this study, IL‐8 concentration peaked at 6 h after talc stimulation. Acencio et al. (2007) investigated the reaction of rabbit pleural mesothelial cells to in vitro talc stimulation and showed, that IL‐8 concentration increased in a time‐dependent manner. Although in our study there was a trend to increase IL‐6 and IL‐8 protein levels in cultures stimulated with all sclerosants (in both doses) after 24 h of exposure compared to cell cultures without stimulation and to their levels 6 h after exposure, the differences were not statistically significant. The small sample size in our study and the potential impact of the different nature of animal and human cells and their reactions to sclerosing agents should certainly be taken into account when considering our findings together with those reported by Acencio et al.

Interestingly, a trend to increase in IL‐6 mRNA expression 6 and 24 h after exposure to the lower TGF‐β dose was demonstrated. According to one previous study, TGF‐β pleurodesis was not associated with an acute inflammatory response (Gary Lee et al., 2003). Our single observation does not empower any binding conclusions, but requires further investigation.

In this study, TGF‐β was not only used as a sclerosing agent but also investigated as a molecule potentially produced by mesothelial cells which might be involved in pleurodesis. This growth factor is known to modulate inflammatory processes. In pleurodesis, it acts as a chemoattractant for fibroblasts and shows profibrotic properties. In the animal study by Marchi et al., TGF‐β concentration increased after talc stimulation (Marchi et al., 2006). The in vitro study by Acencio et al. showed an initial increase in TGF‐β after talc stimulation and its stable level after 48 h (Acencio et al., 2007). In our study, there was a trend to increase TGF‐β mRNA expression after 24 h in groups stimulated with doxycycline (both doses), PVP‐I and talc (lower doses), however, the differences were not statistically significant. The effect was not visible with higher doses of PVP‐I and talc. We cannot exclude that the mesothelial cells used in our experiment could have been more sensitive to PVP‐I concentrations that were earlier used and adopted for our study (Fiorelli et al., 2014).

A significant role of MCP‐1 in pleural fluid formation (especially MPE) has been postulated (Thomas et al., 2016). The blockage of MCP‐1 resulted in a significant decrease in pleural effusion volume in an animal model of benign pleurisy induced by λ‐carrageenan (Lansley et al., 2017). In our study, we did not observe any significant change in MCP‐1 expression in response to stimulation with any sclerosant.

Our study has several limitations. First, due to the exploratory nature of the study, it was performed on a relatively small sample. Moreover, the cultures selected for the essential experiments included pleural mesothelial cells isolated from one patient only. Hence, the results may reflect the individual features of pleural mesothelial cell responses. As the patient had no history of pleural disease, the pleural mesothelial cells were obtained from a different milieu than that in pleura involved in the malignant process. It is therefore important to realize that the responses found in our model may not necessarily be the same as those presented by pleural mesothelial cells in patients with MPE. However, it should be emphasized that the isolation and propagation of pleural cells is demanding, and therefore most of the research in this subject has been carried out on immortalized cells. Our study is one of the few performed on the primary pleural cell line. Second, we did not perform standardization for cell numbers other than relative confluence which could have had an impact on the protein concentration measurements. This may constitute a methodological shortcoming. In the case of PCR, the 2^−∆∆CT^ method was used, which optimizes this error due to self‐reference with the endogenous gene mRNA expression. Third, all used reagents were free of endotoxins except for PVP‐I. As this report is looking at inflammatory cytokines, results with this sclerosant should be interpreted more cautiously.

Additionally, we used a relatively simple, mono‐cell culture experiment. As pleurodesis is a complex process involving many types of cells, this experiment may be impoverished and may not reveal all mechanisms, including the complicated and subtle cell‐cytokine interplay. Nevertheless, this study can be regarded as an important step to develop more complex multi‐cell 3D cultures that may be a more realistic model for studying the mechanisms of chemical pleurodesis.

## 5 CONCLUSIONS

Our study confirmed that culture of pleural mesothelial cells isolated from pleura biopsy specimens is feasible. These cells responded to stimulation with different sclerosing agents and this response could be measured as the changes in mRNA expression for various cytokines and growth factors, as well as the changes in the level of these biomarkers. The responses were highly variable and there were no consistent patterns of mesothelium reaction neither in terms of different sclerosing agents nor in the time of the most significant reaction. This study may suggest the role of IL‐1β in the formation and maintenance of the inflammatory response during pleurodesis. We believe further in vitro studies on the mechanisms of pleurodesis should involve more complex, two‐ or three‐cell‐type cultures that may better reflect the natural pleural milieu.

## CONFLICT OF INTEREST

The authors declare no conflicts of interest in relation to this article.

## AUTHOR CONTRIBUTION

Mierzejewski M. and Paplińska‐Goryca M designed the study, carried out the isolation and culture of cells, conducted laboratory assays, analyzed data, and wrote the first draft of the manuscript. Korczyński P. designed the study and carried out sample acquisition. Krenke K. designed the study and edited the manuscript. All authors critically reviewed the manuscript and contributed to the final version. All authors have read and agreed to the published version of the manuscript.

## Data Availability

The data that support the findings of this study are available from the corresponding author upon reasonable request.
